# Root discrimination of closely related crop and weed species using FT MIR-ATR spectroscopy

**DOI:** 10.3389/fpls.2015.00765

**Published:** 2015-09-29

**Authors:** Catharina Meinen, Rolf Rauber

**Affiliations:** Division of Agronomy, Department of Crop Sciences, Georg-August-University Goettingen, GoettingenGermany

**Keywords:** cluster analysis, root discrimination, species proportion, spectral distribution, chemical root composition

## Abstract

Root discrimination of species is a pre-condition for studying belowground competition processes between crop and weed species. In this experiment, we tested Fourier transform mid-infrared (FT MIR)-attenuated total reflection (ATR) spectroscopy to discriminate roots of closely related crop and weed species grown in the greenhouse: maize/barnyard grass, barley/wild oat, wheat/blackgrass (Poaceae), and sugar beet/common lambsquarters (Chenopodiaceae). Fresh (moist) and dried root segments as well as ground roots were analyzed by FT MIR-ATR spectroscopy. Root absorption spectra showed species specific peak distribution and peak height. A clear separation according to species was not possible with fresh root segments. Dried root segments (including root basis, middle section, and root tip) of maize/barnyard grass and sugar beet/common lambsquarters formed completely separated species clusters. Wheat and blackgrass separated in species specific clusters when root tips were removed from cluster analysis. A clear separation of dried root segments according to species was not possible in the case of barley and wild oat. Cluster analyses of ground roots revealed a 100% separation of all tested crop and weed species combinations. Spectra grouped in Poaceae and Chenopodiaceae clusters. Within the Poaceae cluster, C_3_ and C_4_ species differed significantly in heterogeneity. Thus, root spectra reflected the degree of kinship. To quantify species proportion in root mixtures, a two- and a three-species model for species quantification in root mixtures of maize, barnyard grass, and wild oat was calculated. The models showed low standard errors of prediction (RMSEP) and high residual predictive deviation values in an external test set validation. Hence, FT MIR-ATR spectroscopy seems to be a promising tool for root research even between closely related plant species.

## Introduction

Competition processes between agricultural crops and weeds are a challenging field in crop science. Crop and weed species often compete for the same resources such as nutrients, water, and light (Zimdahl, 2004). Many former studies about crop–weed interactions focused only on aboveground competition or used aboveground parameters to explain the impact of the competition for water and nutrients. However, competition should be explained at a whole plant level (Rajcan and Swanton, 2001). Plants within one plant family are often similar in their strategy to acquire nutrients and water which can increase the competition. In several studies, root competition was more intense than shoot competition (Martin and Field, 1987; Satorre and Snaydon, 1992; Casper and Jackson, 1997; Kiaer et al., 2013). In general, studies of belowground competition between crop and weed species are scarce, often due to the difficulties of studying the species specific root systems in mixtures.

*Echinochloa crus-galli* (barnyard grass), *Alopecurus myosuroides* (blackgrass), *Avena fatua* (wild oat), and *Chenopodium album* (common lambsquarters) are common weed species in Central Europe and can account for high yield losses, e.g., in maize up to 82% (Spitters et al., 1989), and in winter wheat (Naylor, 1972). Lambsquarters species and barnyard grass show wide distribution and high plant densities, e.g., in maize (Mehrtens et al., 2005), and barley fields (Williams, 1963). Common lambsquarters, also a frequent weed in sugar beet, has been used as a competitive weed in a mechanistic crop–weed model (Kropff and Spitters, 1992). To expand and improve crop–weed models, more detailed information about root growth and response to interspecific competition is essential. For example in sugar beet, breeding lines with a high root length density showed high productivity and low weed densities indicating a strong competitive ability (Stevanato et al., 2011). Like for most crop and weed species, root investigations in the lifecycle of crop species and associated weeds are still lacking (Rajcan and Swanton, 2001). One reason is that especially belowground competition is difficult to quantify (Kropff and van Laar, 1993).

To analyze belowground competition processes of crop and weed species, roots have to be distinguished according to species. Up to now, a reliable and easy method for plant root discrimination does not exist for plant species. Generally, roots of species within one plant family are quite similar in morphology, texture and color and cannot be distinguished from one another only by visually inspection, e.g., in soil cores or at profile walls. Different approaches were made to study roots in mixtures (Rewald et al., 2012). Some methods based on labeling roots with recognizable substances such as stable isotopes (Jensen, 1996; Hauggaard-Nielsen et al., 2009), radioisotopes as ^32^P (Hauggaard-Nielsen et al., 2001) or natural differences in ^13^C:^12^C isotope ratios in C_3_ and C_4_ plants (Gealy and Fischer, 2010). Corre-Hellou and Crozat (2005) utilized herbicide injections and ^15^N natural abundances to study rooting depth and root biomass contribution of pea, barley, and mustard in mixtures. On a molecular level, Linder et al. (2000) developed a DNA-based method to identify roots to the level of genus. Mommer et al. (2008) improved this technique and presented a quantitative molecular procedure to discriminate and quantify roots in species mixtures, which is unaffected by a changing soil environment. As a pre-condition, DNA of all species has to be extracted from sole crops or has to be available in reference databases. The calibrations for the quantitative analyses are extensive and many chemicals are needed. Lei and Bauhus (2010) predicted the species proportions in root mixtures of four tree species by near infrared reflectance spectroscopy. To profit from the chemical differences in roots of species, Fourier transform mid-infrared (FT MIR) attenuated total reflection (ATR) spectroscopy was used to distinguish successfully single root segments of pea and oat roots growing sole, in mixtures and under different soil conditions (Naumann et al., 2010).

Fourier transform mid-infrared-attenuated total reflection spectroscopy irradiates a sample with mid-infrared light waves which penetrates it only a few micrometer in depth (Clarke et al., 2002). The chemical composition of a sample determines the spectral pattern as a function of wavenumber (Chalmers and Griffiths, 2002; Günzler and Gremlich, 2002). Thus, FT MIR-ATR spectra can be used as spectral fingerprints and the method was already used for species discrimination of bacteria and fungi (Naumann et al., 1991, 2005; Mariey et al., 2001; Naumann, 2009). FT MIR-ATR spectroscopy offers many advantages besides the highly characteristic fingerprint region, such as low sample preparation (drying, grinding), low maintenance costs, no chemical wastage, and short measuring times due to the ATR device. Only a small amount of each sample material is needed to record a spectrum which is a helpful benefit in the case of roots.

In this study, we tested FT MIR-ATR spectroscopy to discriminate roots of closely related crop and weed species: maize/barnyard grass, barley/wild oat, wheat/blackgrass, and sugar beet/common lambsquarters. Furthermore, a model for species quantification in root mixtures of two (maize, wild oat) and three species (maize, wild oat, barnyard grass) mixtures was developed and validated with an external test set.

## Materials and Methods

### Plant Material

In a greenhouse experiment in 2011, crops and weed species were grown sole and in mixtures. The combinations of the Poaceae plant family were: maize (*Zea mays* L.), cultivar “Ricardinio” and barnyard grass (*Echinochloa crus-galli* L.), provenance Goettingen, barley (*Hordeum vulgare* L.), cultivar “KWS Bambina” and wild oat (*Avena fatua* L.), provenance Goettingen, spring wheat (*Triticum aestivum* L.), cultivar “KWS Chamsin” and blackgrass (*Alopecurus myosuroides* Huds.), provenance Goettingen. As Chenopodiaceae species, sugar beet (*Beta vulgaris* subsp. *vulgaris* var. *altissima* Döll.), cultivar “Isabella KWS” and common lambsquarters (*Chenopodium album* L.), provenance Goettingen, were cultivated. Plants were sown in a combination of sand and compost (50 vol.%–50 vol.%) in single species pots (11 cm × 11 cm) with two plants of one species per pot, and in mixtures with one crop and one weed species per pot (**Table 1**). Both, the single species and the mixture species pots were used for the cluster analysis of the fresh and dried root segments. Furthermore, the total root mass of the single species pots were used for the cluster analysis of the ground roots and the building of the quantification model. Three replicates of crop/weed-combinations in single species and mixture pots were randomly distributed within the greenhouse. Plants were cultivated under natural light conditions from March to May 2011. Maize was harvested at growth stage BBCH 15–16 (Meier, 2001), barnyard grass at BBCH 23, barley at BBCH 26–31, wild oat at BBCH 24–31, wheat at BBCH 24–31, blackgrass at BBCH 29, sugar beet at BBCH 27–28, and common lambsquarters at BBCH 59–63. Air and soil temperature averaged at 20°C during the growing period. Due to ambiguity a second barley cultivar was tested in a comparable greenhouse experiment in 2013 with barley (*Hordeum vulgare* L.), cultivar “Marthe” and wild oat (*Avena fatua* L.), provenance Goettingen.

**Table 1:** Combinations of crop and weed species in pots, BBCH growth stage at harvest, date of harvest, and number of replicates.

Species combination	BBCH stage at harvest	Date of harvest	Replicates	
			Root segments	Ground roots
Maize/maize	15–16	2 May, 2011	3	6
Barnyard grass/barnyard grass	23	2 May, 2011	3	6
Maize/barnyard grass	15–16/23	2 May, 2011	3	–
Wild oat/wild oat	24–31	6 May, 2011	3	6
Barley/barley (KWS Bambina)	26–31	6 May, 2011	3	6
Barley (KWS Bambina)/wild oat	24–31/26–31	6 May, 2011	3	–
Wheat/wheat	24–31	11 May, 2011	3	6
Blackgrass/blackgrass	29	11 May, 2011	3	6
Wheat/blackgrass	24–31/29	11 May, 2011	3	–
Sugar beet/sugar beet	27–28	24 May, 2011	3	6
Common lambsquarters/common lambsquarters	59–63	24 May, 2011	3	6
Sugar beet/common lambsquarters	27–28/59–63	24 May, 2011	3	–
Wild oat/wild oat	26–31	1 July, 2013	3	6
Barley/barley (Marthe)	28–31	1 July, 2013	3	6
Barley (Marthe)/wild oat	28–31/26–31	1 July, 2013	3	–

Roots of harvested plants were rinsed with a soft water-jet to remove soil particles. Six root segments per plant were cut off and placed in 2 ml reaction tubes. Two root segments of 1 cm length were collected at the basis of the root system, in the middle section of a root and at root tips. Additionally, taproot segments were collected from sugar beet and common lambsquarters. The fresh (moist) root segment was measured immediately by FT MIR-ATR spectroscopy. The second of the two root segments was dried at 50°C for 48 h before spectra were recorded by FT MIR-ATR spectroscopy according to Naumann et al. (2010). The total root mass of the single species pots were dried at 50°C for 48 h, ground to 0.2 mm (centrifugal mill, Retsch, ZM 100) and subjected to FT MIR-ATR spectroscopy.

### FT MIR-ATR Spectroscopy

Spectral analysis was accomplished by a FT MIR-ATR spectrometer (Alpha, Bruker Optics, Ettlingen, Germany) with an ATR device (diamond crystal). The root segments were placed on top of the ATR crystal, the infrared beam is totally reflected at the interface between the sample and the crystal. At the interface, the radiation interacts with the sample and is attenuated. Spectra were recorded with a resolution of 4 cm^-1^ and 64 scans in the spectral wavenumber range of 4000–400 cm^-1^. The FT MIR-ATR spectrum is calculated from the attenuated beam and displayed as absorbance against wavenumber (cm^-1^).

The spectra were tested for their similarity by cluster analyses (software OPUS, version 7.0, Bruker Optics, Ettlingen, Germany). Therefore, spectra were pre-processed by calculating the first derivative, vector normalization and offset-correction. The first derivative emphasizes steep edges of a peak. It is used to bring out pronounced, but small features over a broad background. The vector normalization normalizes a spectrum by first calculating the average intensity value and subsequently subtracting this value from the spectrum. Then the sum of the squared intensities is calculated and the spectrum is divided by the square root of this sum. This method is used to account for different sample thicknesses (Bruker, 2011; Hanson, 2015). The offset-correction shifts the spectra in order to set the *y*-minimum to zero (Bruker, 2011). The spectral ranges of 3751–2749 and 1800–599 cm^-1^ showed distinct peak differences and therefore were used to construct cluster dendrograms by means of Ward’s algorithm and Euclidean distance (Bruker, 2011). The Ward’s algorithm belongs to the hierarchical clustering methods (Varmuza and Filzmoser, 2009) and tries to find as homogeneous groups as possible (Bruker, 2011). Only two groups are merged which show the smallest growth in heterogeneity factor *H*. *H*(*r*,*i*) is calculated according the following equation:

$$H\,\left(r,\;i\right)=D\,\left(r,\;i\right)=\frac{\left[n\left(p\right)\;+\;n\left(i\right)\right]\;*\;D\,\left(p,\;i\right)\,\;+\;\left[n\left(i\right)\;+\;n\left(q\right)\right]\;-n\left(i\right)\;*\;D\,\left(q,\;i\right)\,*D\,\left(q,\;i\right)}{n\,+\;n\left(i\right)}$$

The *p* and *q* clusters are merged to the new *r* cluster. *D*(*r*,*i*) is the spectal distance between the new *r* and the *i* clusters. *D*(*p*,*i*) and *D*(*q*,*i*) are the spectral distances between the *p* and the *i* clusters and the *q* and the *i* clusters. *n* is the total number of reference spectra. *n*(*p*), *n*(*i*), and *n*(*q*) are the number of spectra which are merged in the *p*, *i*, and *q* clusters (Bruker, 2011).

### Quantification Model

The following species quantification model by FT MIR-ATR spectroscopy is derived from species quantification by FT NIR spectroscopy described by Lei and Bauhus (2010). Thereby, Lei and Bauhus (2010) determined the species specific fine root proportion in root mixtures of up to four tree species and non-woody plants.

We used ground root mass of sole grown maize, wild oat, and barnyard grass to calibrate and validate a model for species quantification in root mixtures of unknown species compositions. The 2-species model with 21 artificial root mixtures was prepared in 5% steps from 0 to 100% of maize and wild oat, respectively. A second model with three species included maize, wild oat, and barnyard grass and was calibrated with 21 artificial root mixtures. Root mixtures contained 15–80% maize, 1.5–79% wild oat, and 0.5–20% barnyard grass. Prepared mixtures were recorded three times by FT MIR-ATR-spectroscopy. Model development and data analysis were carried out with the software OPUS QUANT 2 (Version 7.0, Bruker, 2011). The algorithm of the OPUS QUANT 2 software option is not publicly available but is derived from the method of Lei and Bauhus (2010). A cross-validation option was used to build the model. The validation training excludes one sample from the calibration set and tests with this excluded sample the predictive power of the model. This is a standard procedure (Varmuza and Filzmoser, 2009) and is repeated until all samples in the data set are used once for the validation (Bruker, 2011). The best model with the highest coefficient of determination (*R*^2^) and the lowest root mean square error of cross validation (RMSECV) was chosen by running a procedure for model optimization provided by the software. *R*^2^, RMSECV, spectra pretreatments, and spectral ranges are shown in **Table 2**. Additionally, an independent sample set, which was not included in the model calibrations, with known species proportion was taken into account for an external validation (**Table 2**).

**Table 2:** Statistical parameters of Fourier transform mid-infrared-attenuated total reflection (FT MIR-ATR) model in terms of calibration and internal cross validation.

Model	Calibration				Internal cross validation			
	*n*	*R*^2^	RMSEE	RPD	*R*^2^	RMSECV	Bias	RPD
**2-Species**								
Maize	21	0.99	2.65	12.1	0.99	3.48	0.20	8.73
Wild oat	21	0.99	2.65	12.1	0.99	3.48	–0.20	8.73
**3-Species**								
Maize	23	0.98	4.00	6.67	0.96	5.12	–0.11	5.05
Wild oat	23	0.98	3.93	7.11	0.97	4.91	0.15	5.51
Barnyard grass	23	0.98	1.67	6.66	0.91	3.16	0.14	3.36

*Model quality is described by coefficient of determination (*R*^2^), root mean square error of calibration (RMSEE), root mean square error of cross validation (RMSECV), and residual predictive deviation (RPD). 21 and 23 spectra (*n*) were recorded three times for the calibration of the 2- and 3-species model, respectively. Spectra for the 2-species model were vector-normalized and integrated in the calibration model in the range of 3998-3635, 3274-2186, 1825-1460, and 1099-735 cm^-1^. For the 3-species model calibration, spectra were pre-treated by calculating the first derivative and multiplicative scattering correction with 17 smoothing points in the range of 3274-2910 and 1825-735 cm^-1^. An optimization procedure was used to select a model with the lowest RMSECV.*

## Results

### Spectral Patterns of Roots

The spectra of fresh root segments showed very similar peak distributions and heights in the whole wavenumber range of 4000–400 cm^-1^ (data not shown), while spectra of dried root segments and ground roots showed only similar peak distribution between wavenumber 4000 and 1800 cm^-1^.

**FIGURE 1:**
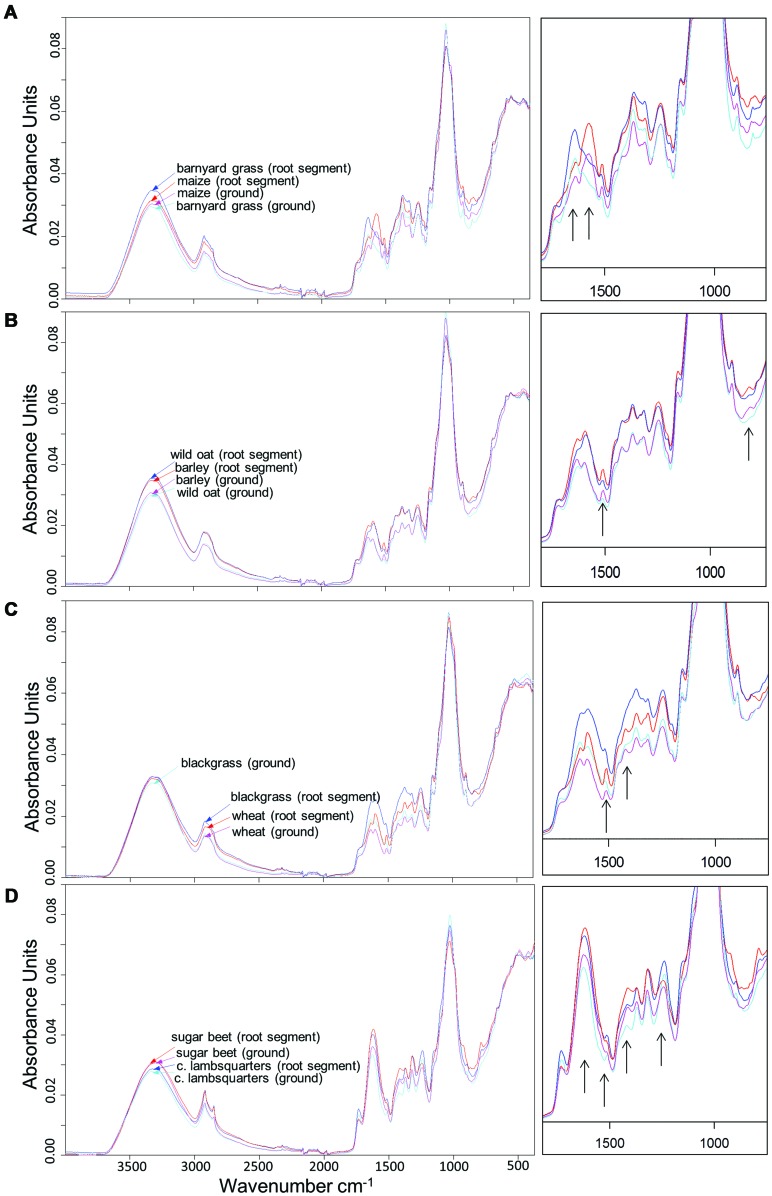
**Fourier transform mid-infrared-attenuated total reflection (FT MIR-ATR) spectra of dried root segments and ground roots from **(A)** maize and barnyard grass, **(B)** barley (cultivar KWS Bambina), and wild oat, **(C)** wheat and blackgrass, and **(D)** sugar beet and common lambsquarters.** Spectra were vector-normalized and offset-corrected. Spectra of rootlets were means of 27 spectra (root tips, middle section, and root basis) and ground root spectra of each species were means of six spectra (plant root biomass of intraspecific pots). The part of 1800–700 cm^-1^ is magnified and black arrows point to species specific peaks.

All dried root segments and ground roots demonstrated peaks at 3330, 2921, and 1030 cm^-1^. From 1800 to 800 cm^-1^, peak distribution and height differed among species. Highest absorbance rates were found around 1030 cm^-1^ (**Figure 1**). While peak distribution varied between species, spectral differences of dried root segments and ground roots within a species were low. Dried root segments and ground roots of maize and barnyard grass showed similar peak distribution and peak height except in the range of 1800 and 1200 cm^-1^ (**Figure 1A**). In this range maize roots exhibited a distinct peak at 1572 cm^-1^ while barnyard grass showed a peak at 1637 cm^-1^. Barley (KWS Bambina) and wild oat root segments as well as ground roots showed very similar peak locations, but dried root segments of both species exhibited higher absorbance rates than ground roots between 3600 and 1200 cm^-1^ (**Figure 1B**). Dried root segments and ground roots of barley held distinct peaks at 1510 and 815 cm^-1^. Dried root segments and ground roots of wheat and blackgrass displayed very different peak heights between 1800 and 1200 cm^-1^ (**Figure 1C**) and wheat roots showed peaks at 1510 and 1420 cm^-1^. Absorbances of dried root segments of blackgrass were higher than those of dried root segments of wheat in the range of 1800–1200 cm^-1^. Spectra of sugar beet and common lambsquarters root segments and ground roots exhibited similar peak location but differed in peak height. Dried root segments of sugar beet showed highest absorbance at 1621 and 1416 cm^-1^ while dried root segments of common lambsquarters peaked at 1516 and 1240 cm^-1^ (**Figure 1D**).

### Root Segments Discrimination by Cluster Analysis

The heterogeneity of root segments spectra was tested by cluster analyses. The spectral patterns of fresh roots were very similar for the species and cluster analysis revealed no clear separation between species (data not shown).

**FIGURE 2:**
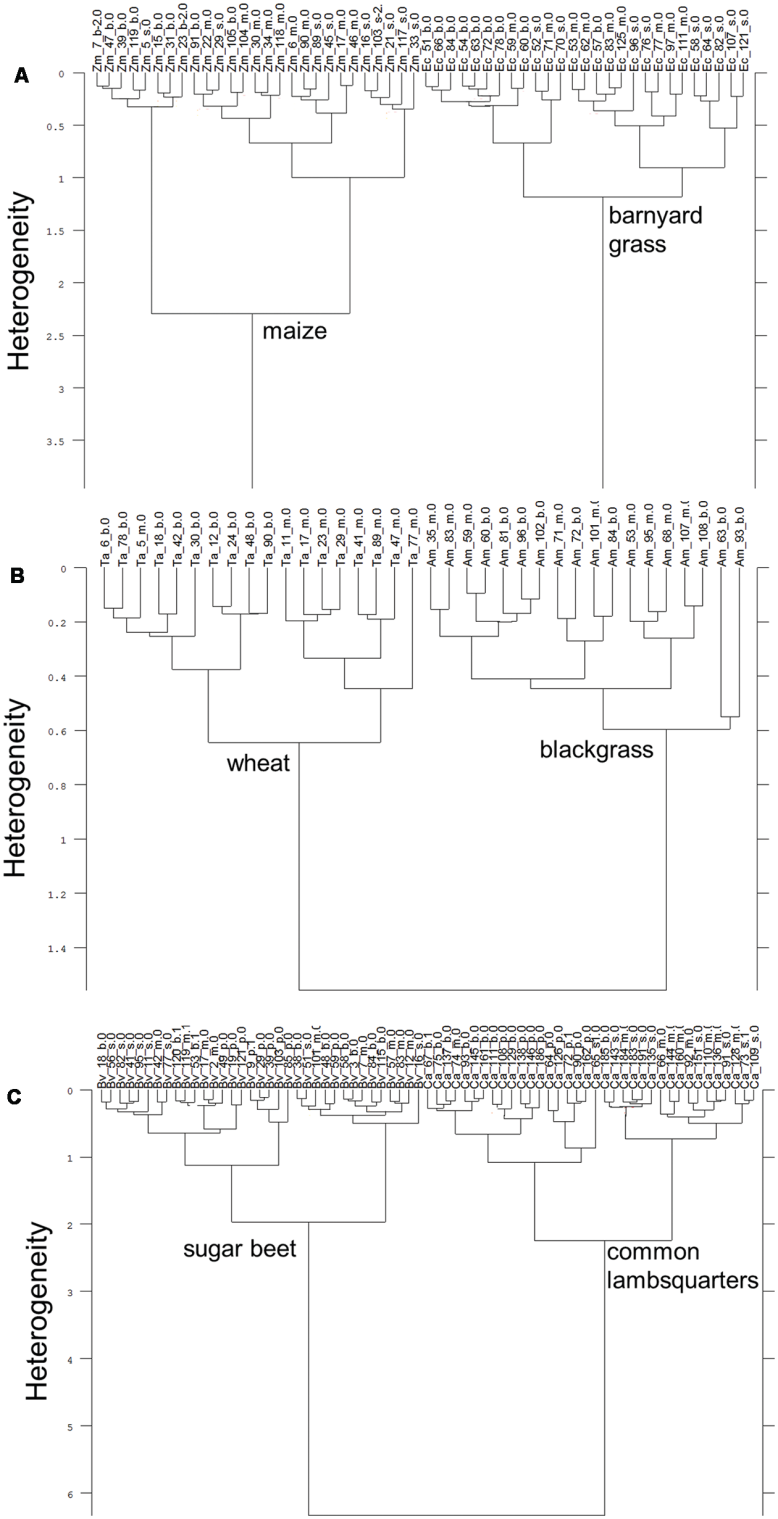
**Cluster analysis of FT MIR-ATR spectra recorded from dried root segments of **(A)** maize (Zm) and barnyard grass (Ec), **(B)** wheat (Ta) and blackgrass (Am), and **(C)** sugar beet (Bv), and common lambsquarters (Ca).** Root segments are taken from root basis (b), mid-section (m), and root tip (s). Analysis included three replicates. Tap root segment (*p*) were also measured for sugar beet and common lambsquarters. Spectra were pre-processed by first derivative and vector normalization. Ward’s algorithm was applied in the frequency range of 3751–2749 and 1800–599 cm^-1^.

Dried root segments of maize and barnyard grass clearly split up into species specific subclusters (**Figure 2A**). Intraspecific heterogeneity in maize was 2.29 and 1.18 in barnyard grass. Interspecific heterogeneity between species was 3.97 which is 1.7 times higher than intraspecific heterogeneity of maize and 3.4 times higher than intraspecific heterogeneity of barnyard grass. Some of the root basis segments and one tip segment of maize constituted a subcluster within the maize cluster which increased the heterogeneity of the whole maize cluster. A clear separation of dried root segments according to species was not possible in the case of both barley cultivars (KWS Bambina and Marthe) and wild oat (data not shown). Dried root segments of wheat and blackgrass separated into species specific clusters when root tips were excluded from the analysis. Intraspecific heterogeneity was 0.65 in wheat and subclusters of root basis and middle sections were formed (one exception in root basis subcluster) while blackgrass spectra joined at 0.60 (**Figure 2B**). Both clusters joined at 1.56 which is 2.4 times higher than intraspecific heterogeneity of wheat and 2.6 times higher than intraspecific heterogeneity of blackgrass. Dried root segments of sugar beet and common lambsquarters show a clear separation according to species (**Figure 2C**). The intraspecific heterogeneity of sugar beet was 1.97 and somewhat lower than the intraspecific heterogeneity of common lambsquarters (2.24). The interspecific heterogeneity was 6.34 and considerably higher than the intraspecific heterogeneity of sugar beet and common lambsquarters. There was no spectral difference between the dried root segments of the single species pots and the mixture species pots.

### Ground Root Discrimination by Cluster Analysis

**FIGURE 3:**
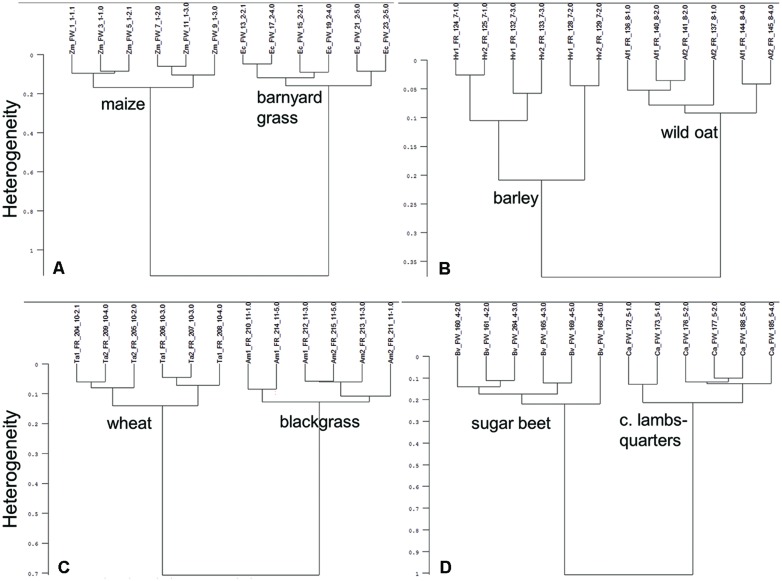
**Cluster analysis of FT MIR-ATR spectra recorded from ground roots of **(A)** maize (Zm) and barnyard grass (Ec), **(B)** barley (Hv), cultivar KWS Bambina and wild oat (Af), **(C)** wheat (Ta) and blackgrass (Am), and **(D)** sugar beet (Bv) and common lambsquarters (Ca).** Spectra were preprocessed by first derivative and vector normalization. Ward’s algorithm was used within the frequency range of 3751–2749 and 1800–599 cm^-1^.

Cluster analysis showed that ground roots of tested crop and weed species combinations are split-up into species specific clusters (**Figure 3**). Maize and barnyard grass divided clearly into two clusters. Intraspecific heterogeneity in maize was 0.17 and 0.16 in barnyard grass, whereas interspecific heterogeneity amounted to 1.13 (**Figure 3A**). Root spectra of barley, cultivar KWS Bambina, and wild oat separated into species specific clusters. Intraspecific heterogeneity of barley was high (0.21) compared to wild oat (0.09). Interspecific heterogeneity of barley (KWS Bambina) and wild oat was only 0.39 (**Figure 3B**). Root spectra of barley, cultivar Marthe, and wild oat separated also into species specific clusters (data not shown). Intraspecific heterogeneity of wheat and blackgrass were 0.13 and 0.12, respectively (**Figure 3C**). Interspecific heterogeneity of wheat and blackgrass was 0.69. Intraspecific heterogeneity was 0.19 in sugar beet and 0.20 in common lambsquarters while interspecific heterogeneity was 0.99 (**Figure 3D**). In all crop–weed combinations except barley (KWS Bambina) and wild oat, interspecific heterogeneity was at least 5.0 times higher than intraspecific heterogeneity showing clear species discrimination based on root spectra.

**FIGURE 4:**
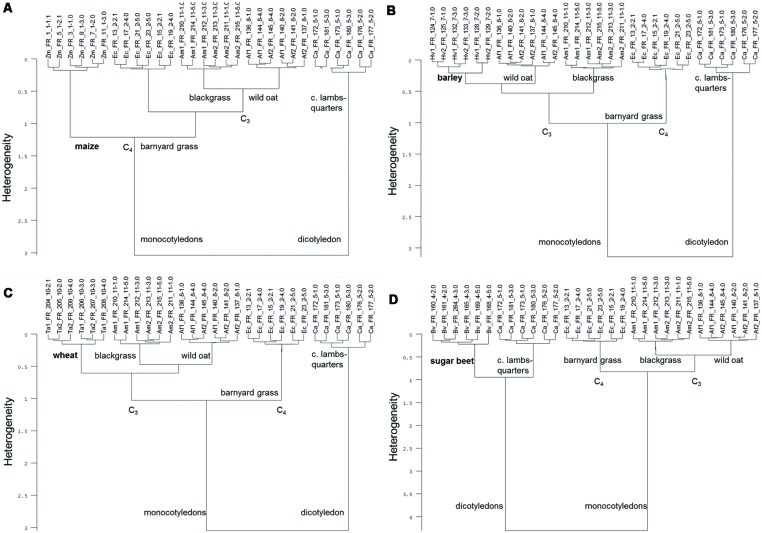
**Cluster analysis of FT MIR-ATR spectra recorded from ground roots of each crop [maize (Zm), barley (Hv), cultivar KWS Bambina, wheat (Ta), sugar beet (Bv)] and the weed species barnyard grass (Ec), wild oat (Af), blackgrass (Am), and common lambsquarters (Ca).** Cluster analysis of ground roots of **(A)** maize, **(B)** barley, **(C)** wheat, and **(D)** sugar beet with all tested weed species. Spectra were preprocessed by first derivative and vector normalization. Ward’s algorithm and Euclidean distance was used within the frequency range of 3999–374 cm^-1^ **(A,D)**, 3663–2776 cm^-1^ and 1846–591 cm^-1^ **(B)**, and 3982–391 cm^-1^ **(C)**.

The cluster analysis of the ground roots of each crop species with all tested weed species revealed a 100% species-specific grouping (**Figure 4**). These analyses showed not only species-specific cluster building, but also reflect the relationship between species: in the analysis of maize, barley, wheat, and sugar beet, two main clusters are clearly formed by monocotyledonous and dicotyledonous species (**Figures 4A–D**). Within the monocotyledonous species clusters, the C_4_ species showed a higher heterogeneity than the C_3_ species (**Figures 4A–D**).

### Quantification Model

The statistical calibration parameters of the 2- and the 3-species models showed mostly high model quality in calibration and validation with low root mean square errors of estimation (RMSEE) and cross validation (RMSECV), and high residual predictive deviation (RPD) values (**Table 2**). In the 2-species calibration model, the coefficient of determination (*R*^2^) was higher (0.99) than in the 3-species model (0.98). The RMSEE was 2.65 in the 2-species calibration model, while RMSEE in the 3-species calibration model was lower in barnyard grass (1.67) and higher in wild oat (3.93) and maize (4.00). RPD was excellent with 12.1 in the 2-species calibration model and very good in the 3-species calibration model ranging from 6.7 to 7.1.

The *R*^2^ of the internal cross validation were lower compared to the calibration and ranged from 0.91 for barnyard grass in the 3-species model to 0.99 for maize and wild oat in the 2-species model. The RMSECV for maize and wild oat was lower in the 2- than in the 3-species model.

**FIGURE 5:**
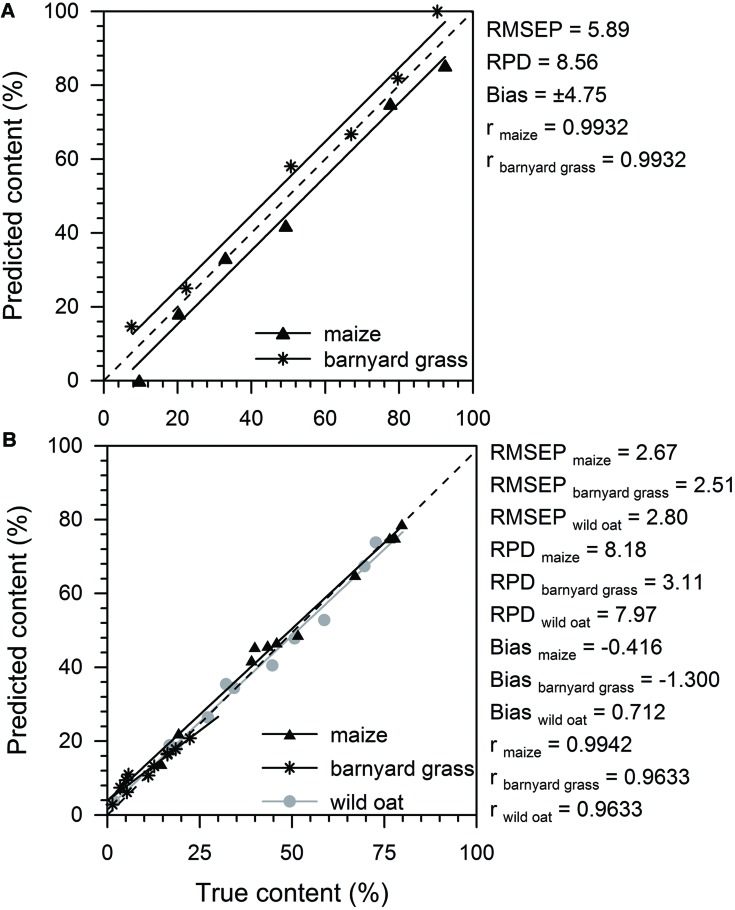
**External test set validation parameters and predicted (%) versus true content (%) of maize and barnyard grass of a 2-species model **(A)** and maize, barnyard grass, and wild oat of a 3-species model (B).** Model quality is described by root mean square error of prediction (RMSEP), bias, residual predictive deviation (RPD), and the correlation coefficient (*r*). The external test set validations were performed with 6 and 11 spectra for the 2- and 3-species model, respectively. Black triangles (maize), black asterisks (barnyard grass), and gray dots (wild oat) represent the predicted versus the true content of the tested components. Linear regression lines (black = maize, barnyard grass; gray = wild oat) are shown. The dashed line identified the rated values.

The external test set validation revealed low RMSEP values and high RPD values (**Figure 5**). The RPD of 8.56 was very good for the 2-species model and also very good for maize (8.18) and wild oat (7.97) in the 3-species model, but low for barnyard grass (3.11). The predicted maize content tended to be underestimated whereas barnyard grass was overestimated in the 2-species model. Therefore, the bias of ±4.75 was high (**Figure 5A**). In the 3-species model, the predicted maize and wild oat content was in good accordance to the true content and both regression lines were close to the rated values (**Figure 5B**). The predicted barnyard grass content below 10% of true content was overestimated while it was underestimated with increasing content (**Figure 5B**). The bias of maize (–0.416) and wild oat (0.712) were low whereas barnyard grass showed a bias of –1.3 in the 3-species model. Correlation coefficients were high and ranged from 0.96 in barnyard grass in the 3-species model to 0.99 in all other components.

## Discussion

Naumann et al. (2010) demonstrated that FT MIR-ATR spectroscopy can discriminate dried root segments of the far related species pea and oat. The present study showed that successful species discrimination of even closely related species using FT MIR-ATR spectroscopy of dried root segments is possible. Spectra of dried root segments and ground roots demonstrated species specific absorbance peak heights and different peak locations while fresh root spectra were very similar. A complete separation of fresh root segments was not possible. However, three out of four combinations of closely related crop and weed species showed species specific clusters in the cluster analyses of dried root segments. Cluster analyses of ground root spectra revealed not only a 100% separation according to species in every crop–weed combination but also reflected the relationship of the species. Mono- and dicotyledonous species separated into different clusters when ground root spectra were analyzed. Moreover, in the monocotyledonous species, C_3_ and C_4_ species constituted separated clusters. A validated quantification model for two (maize, wild oat) and three (maize, wild oat, barnyard grass) species was developed. The models showed low RMSEP values and high RPD values, except for barnyard grass. The possibility of species identification and quantification using FT MIR-ATR spectroscopy seems to have great potential in future studies to investigate crop and weed species in competition processes, e.g., single root identification and root proportions according to species in mixtures.

### Spectral Differences of Species

Fresh root spectra were very similar in all species and no species specific peaks were revealed. The oscillation of water molecules can cover oscillation of other hydrogen bindings and therefore, the signal of the latter ones is overlapped. This results in spectra which are strongly influenced by the water signals and the significant species specific peaks are concealed (Sherman Hsu, 1997; Allison, 2011). Due to the very similar spectra, the cluster analyses of the fresh root segments revealed no separation according to species. However, fresh roots of distantly related species such as pea and oat grown in the greenhouse could be discriminated (Meinen, unpublished data).

Dried root segments and ground roots showed species specific spectral patterns. All species demonstrated a polysaccharide-associated peak at 1030 cm^-1^ with highest absorbance rates in ground roots of the weed species, especially in the Poaceae species. This is in accordance with Naumann et al. (2010) who found this peak also in pea and oat root segments, mainly with highest absorbance units in the oat roots. This polysaccharide-associated peak lies in the range of 1185–900 wavenumber cm^-1^ and represents cellulose and hemicellulose (Wilson et al., 2000). Furthermore, Naumann et al. (2010) characterized protein- and lipid-associated peaks (1800–1485 cm^-1^ protein, 1485–1185 cm^-1^ protein, lipids) in pea and oat which were a good indicator for species-specific differences. This is analog to findings in this study where we found species specific differences in peak height and location in the range of 1800–600 cm^-1^. Species specific differences in this range were also reported by Rewald et al. (2012) and Rewald and Meinen (2013) for the crop species *Brassica napus* and the weed species *Apera spica-venti* and *Sisymbrium officinale*. Legner et al. (2014) used dried root segments from the distantly related species pea and oat, sampled from a field experiment, identifying successfully the root distribution according to species in a pea–oat-intercropping.

### Cluster Analyses of Dried Root Segments

Cluster analyses of dried root segments revealed a successful species discrimination of maize/barnyard grass and sugar beet/common lambsquarters while a species-specific separation of barley/wild oat was not possible. Even with a second cultivar of barley (cultivar Marthe) a clear separation of dried root segments from barley and wild oat was impossible. The standard deviation of the barley spectra were relatively high compared to the standard deviation of the other species demonstrating a high variation within the barley spectra. A cluster analysis of the dried root basis segments of Poaceae species barley, wild oat, wheat and blackgrass showed that barley spectra were more similar to the weed species wild oat and blackgrass than to wheat. A clear separation of dried root basis segments of barley, wild oat and blackgrass was not possible whereas wheat spectra grouped within one subcluster (data not shown). One explanation for the similarity of the barley spectra to the Poaceae weed spectra on the one hand and their dissimilarity to the wheat spectra on the other hand could be the breeding history of barley and wheat. In contrast to wheat, barley varieties are less formed by breeding and are more similar to older forms of barley than the modern wheat varieties which have only little in common with their origin (Able et al., 2007; Feuillet et al., 2008). Besides this, the diploid barley varieties, as well as wild oat and blackgrass genomes, are much less buffered against genomic perturbations than the hexaploid wheat varieties which possibly make barley more similar to not manipulated or bred species, e.g., weed species (Feuillet et al., 2008).

Spectra of dried wheat and blackgrass root segments grouped according to species when root tips were removed from the analysis. Root tips of these two species seemed to be very similar in their chemical surface composition. In the cluster analyses of maize/barnyard grass and sugar beet/common lambsquarters dried root segments and especially root tips of each species grouped (with exceptions) in subclusters indicating a similar chemical and spectral composition within the segments. Naumann et al. (2010) used FT MIR-ATR spectroscopy to analyze dried roots of pea and oat grown on different substrata and in interspecific competition. They reported that for dried pea and oat root segments, species specific differences were much higher than the influence of substrata or interspecific competition effects on the chemical compositions of the roots. Spectra of dried pea and oat root segments separated totally in species specific clusters (Naumann et al., 2010). The chemical differences of the dried root segments were significantly higher between the species than the influence of interspecific competition in the mixture pots. Thus, there were no spectral differences between the dried root segments of the single species and the mixture species pots. This result is in accordance to findings of Naumann et al. (2010). Naumann et al. (2010) analyzed the carbon (C) and nitrogen (N) content in pea and oat roots and found that the different C/N ratio was also reflected in the FT MIR-spectra (cellulose/protein peak height ratio) of dried pea and oat root segments. Both species are not closely related and it is expected that the chemical composition of a legume and a grass species differ from each other. For closely related species, a distinct difference in the chemical composition is more unlikely. On the one hand, barley and wild oat roots seemed to be very similar in their chemical composition of the surface without species specific characteristics in all dried root segments whereas in wheat and blackgrass, only root tips seemed to be very similar. On the other hand, there are characteristic components in maize and barnyard grass as well as in sugar beet and common lambsquarters roots which are reflected in different peak heights and locations in their species specific root spectrum. The cluster analyses confirmed that even in closely related species, root spectra of dried root segments differed according to species except for barley and wild oat.

### Cluster Analyses of Ground Roots

Species discrimination of ground roots showed a 100% successful separation of all tested combinations according to species. The homogenized root samples evidently increased the heterogeneity in the cluster analyses compared to cluster analyses of dried root segments. Even barley and wild oat spectra separated into species-specific clusters. The beam of a FT MIR-ATR spectrometer penetrates the sample only a few micrometer (0.5–5 μm) in depth (PerkinElmer, 2005). Thus, the zone where a dried root segment is in contact with the evanescent wave is very small. After the process of drying and grinding, the cells were destroyed and the total root mass homogenized. Thus, not only the outer cell layers will be recorded but also cells with more species specific contents. Interspecific heterogeneity of maize/barnyard grass, sugar beet/common lambsquarters, and wheat/blackgrass was 5.0–6.6 times higher than intraspecific species heterogeneity of the species and therefore much higher compared to heterogeneity of dried root segments. Interspecific heterogeneity of barley/wild oat was low (1.8–4.1) compared to intraspecific species heterogeneity, but species discrimination was possible. Zhao et al. (2004) successfully discriminated ground roots of even eight wheat varieties in KBR pellets by FT MIR spectroscopy.

Cluster analyses of each crop species and all tested weed species revealed a 100% species-specific grouping. These analyses revealed not only species-specific clusters, but also reflected the kinship between species in the formation of the subclusters. Phylogenetic relationship of plants was also detected by Kim et al. (2004) via FT MIR spectroscopy. The dendrogram based on PCA of FT MIR data was in accordance to known plant taxonomy. Furthermore, bacteria and fungi, e.g., yeast strains, were successfully discriminated by FT MIR spectroscopy (Mariey et al., 2001; Wenning et al., 2002). In mixed stands, plants species composition can be easily identified above ground, but not belowground. FT MIR spectroscopy can help to distinguish a known species composition also belowground. The identification of species just from the FT MIR-ATR spectra is not possible until there is a reference spectra registered in the spectra library.

### Quantifying Proportion of Species in Root Mixtures

To quantify species proportion in crop and weed species competition processes, we developed a FT MIR-ATR spectroscopy quantification model with two (maize, wild oat) and three (maize, wild oat, barnyard grass) species. The assessment criteria like RPD, RMSEP, and *R*^2^ of the 2- and 3-species models of calibration, validation, and external validation showed that FT MIR-ATR quantification models are a promising tool to quantify root proportions in mixtures of crop and weed species. This is in accordance to similar findings of Rewald and Meinen (2013) in a FT MIR-ATR model for *Vicia faba* and *Matricaria chamomilla*. In many studies, root parameters, e.g., root length density and dry root mass via soil coring is evaluated but the root discrimination between species is usually not possible. Therefore, the combination of soil coring including the standard root parameters with a quantification model on the basis of FT MIR-ATR spectroscopy is possible, even with closely related species, and enables the analysis of root proportions in mixed stands.

Residual predictive deviation values explain the prediction performance of a quantification model. According to Diller (2002), RPD values are sufficient with RPD > 3, good with RPD > 5, and excellent with RPD > 10. The 2-species model (RPD > 8.56) reached a higher precision and accuracy regarding RPD values of the calibration, and the internal and external validation than the 3-species model for maize and wild oat (RPD > 5.05). Barnyard grass showed very low RPD values > 3.11 which are barely sufficient for the prediction performance. This could be due to the sample mixing procedure where barnyard grass is only present in the species proportion with up to 20% (except the pure barnyard grass sample) while maize and wild oat cover species proportions from 0 to 100% in the calibration and external validation samples. The reason for that was the limited root material in barnyard grass. Limited root material of all species in this study effected that the calibration models was developed with only 21 samples in the 2-species model and 23 samples in the 3-species model. The external validation had to be developed with only 6 and 11 samples in the 2- and 3-species models, respectively. Nevertheless, RMSEP values were low and RPD values sufficient. In further studies, it is recommended that sample size should be extended and mixture proportion should cover 0–100% for each species. Extra root material should be provided in greenhouse experiments while there should be no problem in field experiments regarding the root mass. Similar problems were reported by Lei and Bauhus (2010) where a minimum of 500 mg dry root material was necessary to record the spectra for a FT MIR model. In root studies, the amount of root material can be a limiting factor. FT MIR-ATR-spectroscopy offers the possibility to scan only little amounts of dry root material (<10 mg) which is a dramatically reduce of root material compared to FT NIR spectroscopy.

A FT near-infrared (FT NIR) quantification model with 3 to 5 tree species and herb layer roots were developed by Lei and Bauhus (2010) showing also similar values for RPD and *R*^2^ values compared to this study. In contrast to Lei and Bauhus (2010), our results showed a slight decrease in model quality from the 2-species to the 3-species model. In a comparison of quantitative models by NIR- and FT MIR spectroscopy, MIR calibrations had lower root mean errors and higher *R*^2^ values (Reeves, 2010), and lower standard errors of cross-validation, but in most cases, the differences between NIR and MIR calibrations were small (Richardson and Reeves, 2005). Nevertheless, Richardson and Reeves (2005) suggested MIR calibrations as a considerable promise for quantitative analytic work.

## Conclusion

The spectral heterogeneity of the species increased from the fresh to the dried root segments and peaked in the ground roots. The fresh root spectra were too similar to discriminate roots of the closely related species. The spectral differences of the majority of the dried root segments were suitable for the species discrimination. This comprises potential for detailed root competition research of single root segments in distantly and even closely related species mixtures. The spectral heterogeneity was highest in ground roots. The ground root material is the basis of quantification models which can predict root proportions in multi species mixtures as a valuable addition to the standard root parameters.

## Conflict of Interest Statement

The authors declare that the research was conducted in the absence of any commercial or financial relationships that could be construed as a potential conflict of interest.
